# Photostability of plasma polymerized γ-terpinene thin films for encapsulation of OPV

**DOI:** 10.1038/srep45599

**Published:** 2017-03-30

**Authors:** Kateryna Bazaka, Jakaria Ahmad, Michael Oelgemöller, Ashraf Uddin, Mohan V. Jacob

**Affiliations:** 1 School of Chemistry, Physics, and Mechanical Engineering, Queensland University of Technology, Brisbane, QLD 4000, Australia; 2 Electronic Materials Research Lab, College of Science and Engineering, James Cook University, Townsville 4811, Australia; 3 Applied and Green Photochemistry Group, College of Science and Engineering, James Cook University, Townsville 4811, Australia; 4 School of Photovoltaic and Renewable Energy Engineering, University of New South Wales, 2052 Sydney, Australia

## Abstract

Optically transparent, smooth, defect-free, chemically inert and with good adhesion to a variety of substrates, plasma polymers from plant-derived secondary metabolites have been identified as promising encapsulating materials for organic electronics and photovoltaics. Here, we demonstrate that an encapsulating layer of plasma polymerized γ-terpinene reduces degradation-related loss in conversion efficiency in PCPDTBT:PC_70_BM solar cells under ambient operating conditions. The stability of γ-terpinene films was then investigated under extreme UV irradiation conditions as a function of deposition power. When exposed to ambient air, prolonged exposure to UV–A and UV–B light led to notable ageing of the polymer. Photooxidation was identified as the main mechanism of degradation, confirmed by significantly slower ageing when oxygen was restricted through the use of a quartz cover. Under unnatural high-energy UV–C irradiation, significant photochemical degradation and oxidation occurred even in an oxygen-poor environment.

Organic electronic devices promise mechanical flexibility and low-cost fabrication. However, when operating under ambient conditions, organic materials are unstable and require physical encapsulation to protect devices from degradation due to water vapor and oxygen permeation^1^. The use of glass lid encapsulating layers greatly limits the use of these devices in flexible applications, whereas currently-available thin film strategies either require the deposition of multi-layer structures (e.g. Barix barrier layers based on alternating dyads of organic and inorganic films) or rely on slow-throughput atomic layer deposition (ALD)^2^,^3^. This drives an ongoing search for single-layer encapsulating technologies that combine excellent barrier properties with fast, inexpensive fabrication.

Plasma-assisted deposition, e.g. plasma-enhanced chemical vapor deposition or plasma-assisted ALD, enables fast, material- and energy-efficient deposition of environmental barrier films from organic and inorganic precursors directly onto organic optoelectronic devices at near ambient temperature^4^,^5^. The highly-reactive nature of plasmas enables conversion of naturally-occurring, minimally-processed hydrocarbons in liquid, gas, or solid state into a variety of structures, from graphene to polymers^6^. Introduction of plasma polymer thin films analogous to polyethylenimine ethoxylated (PEIE) into polymer solar cells led to up to 20% enhancement in device performance^7^. Over the last several years, thin films from essential oils have been developed and identified as promising candidates for applications in electronics as dielectric and encapsulation layers^8^,^9^,^10^,^11^,^12^,^13^,^14^, and as bio-interface coatings for implantable electronics^4^,^15^,^16^,^17^,^18^. However, to date, the deposition of essential oil-based environmental barriers directly onto organic photovoltaic devices have not been reported.

This study investigates the effect of direct encapsulation of organic photovoltaics, specifically PCPDTBT: PC_70_BM solar cell, by γ-terpinene plasma polymer (pp–GT) using radio frequency (RF) plasma polymerization. Also known as 1-isopropyl-4-methyl-1,4-cyclohexadiene, γ-terpinene belongs to a group of monocyclic terpenes and is isolated directly from *Melaleuca alternifolia* (tea tree) essential oil by distillation^19^. Films of pp–GT are optically transparent in the optical wavelength region, with refractive index of 1.57–1.58 (at 500 nm) and optical band gap (*E*_*g*_) of ~3 eV^10^. Independent of deposition conditions, the surfaces of these polymers are smooth and defect-free, with uniformly distributed morphological features and average roughness well below 0.30 nm^10^.

With the intention to use these films as encapsulation coatings in OPVs, the primary aim of this paper is to investigate the stability of pp–GT film when subjected to UV irradiation. Many common natural and synthetic polymers are subject to UV degradation, with UV rays cleaving tertiary carbon-hydrogen bonds in the chain structures to form free radicals, which are in turn subject to further oxidation by oxygen present in ambient air. This can lead to discoloration, cracking, delamination, with the degree of disintegration linked to the duration and intensity of UV exposure, as well as the presence of heat and oxidizing agents. The chemistry of the polymer, e.g. degree of cross-linking and presence of unreacted monomer, determines the susceptibility of the polymer to UV degradation. Thus, in this study samples fabricated at 10, 25, 50 and 75 W RF power levels are compared to investigate the effect of deposition power on the stability of the polymer films under different irradiation conditions.

## Results

### Stability of encapsulated PCPDTBT:PC_70_BM solar cell

**Figure 1:**
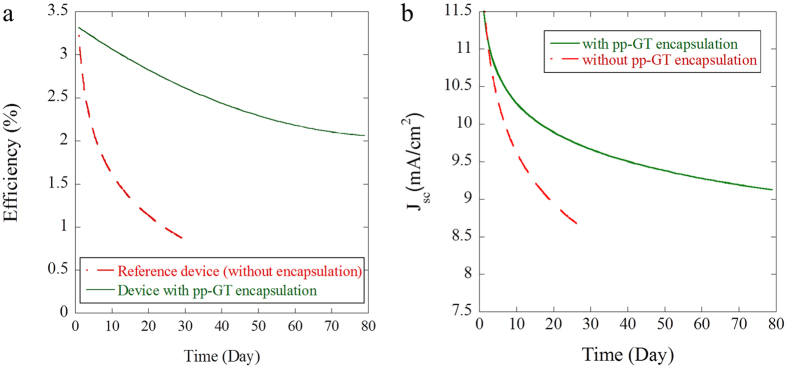
OPV device (**a**) efficiency spectra and (**b**) current density of the reference device and the device with pp–GT thin film encapsulation.

Figure 1 displays the device efficiency spectra of inverted structure PCPDTBT:PC_70_BM solar cells fabricated with and without pp–GT encapsulation. The device with pp–GT thin film encapsulation showed a significantly improved performance when compared to the reference device. The encapsulated and non-encapsulated devices displayed maximum device efficiency value of ~3.3% just after fabrication. Upon exposure to the ambient environment for 1920 h (80 days), the efficiency of encapsulated device gradually decreased, reaching the value of 2.03%. In comparison, the reference device (without encapsulation) experienced a rapid decrease in device efficiency, reaching the value of 1.07% just after 700 h (29 days).

Figure 1 also shows the current density *vs.* time profiles of PCPDTBT:PC_70_BM solar cell fabricated with and without pp–GT thin film encapsulation. The measured short circuit current density follows a similar trend to the device efficiency profiles, reaching a maximum value of 11.52 mA cm^−2^ at the time of device fabrication and decreasing thereafter. For the encapsulated devices, the decrease in short circuit current density was most pronounced within the first 20 days, reaching a value of 8.80 mA cm^−2^ at the end of 80 days. On the other hand, devices within encapsulation experienced a more sharp decrease in the short circuit current density, reaching the value of 8.80 mA cm^−2^ within 17 days and that of 7.96 mA cm^−2^ within 29 days of the exposure. Fill factor (FF) and open circuit voltage (V_oc_) values for the encapsulated device were also significantly higher than those for the reference device. A FF value of 31.89% was measured after 80 days, whereas a value of 28.78% was measured just after 29 days for the reference device.

Dennler *et al*. encapsulated MDMO-PPV:PCBM solar cells with poly(ethylene naphthalate) (PEN)-based barrier material fabricated by PECVD^20^. After 80 days, maximum encapsulated device efficiency was ~60%, which is similar to pp–GT thin film encapsulation architecture. Encapsulated MDMO-PPV:PCBM solar cell had a short circuit current density of ~70% of its initial value after 80 days, whereas pp–GT thin film encapsulation provided ~76% of its initial value. This suggests that encapsulation with pp–GT may be used to extend the lifetime and efficiency of the device. It has also been suggested that the presence of encapsulating layer may positively contribute to the enhancement of the device efficiency related to increased charge carrier mobility and charge carrier lifetime, where the encapsulation reduces the decay rate of photogenerated excitons and improves the efficiency of exciton dissociation^21^.

### Chemical stability of pp-GT films towards UV irradiation

**Figure 2:**
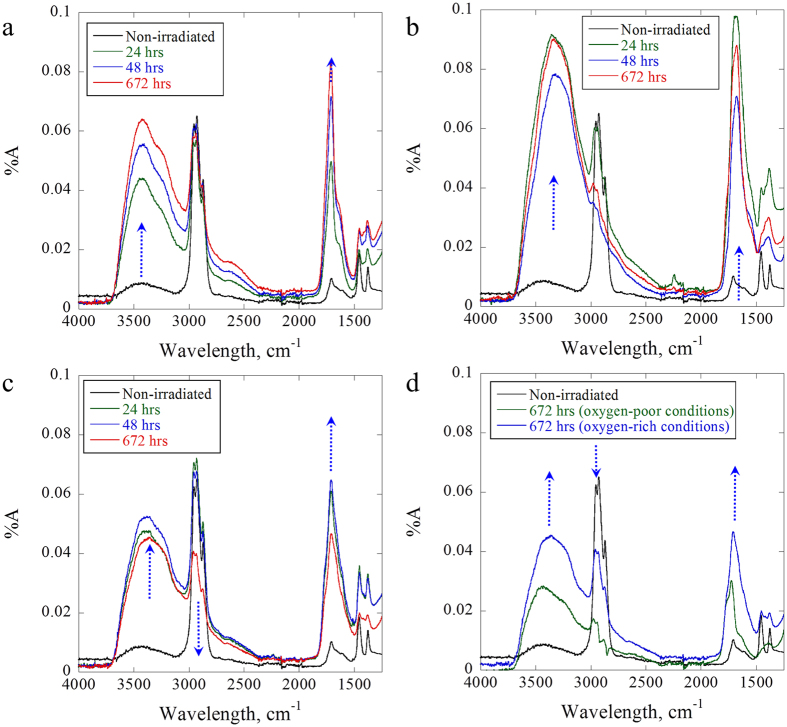
FTIR spectra of samples irradiated with (**a**) UV–A, (**b**) UV–B and (**c**) UV–C under ambient (oxygen-rich) conditions, and (**d**) irradiated with UV–C under oxygen-rich and oxygen-poor conditions. Samples fabricated at 50 W.

In this study, pp–GT thin films have been exposed to UV–A (λ = 350 ± 25 nm), UV–B (λ = 300 ± 25 nm) and UV–C (λ = 254 nm) for 24, 48 and 672 h. Although not naturally occurring, UV–C is commonly used for sterilization or waste treatment purposes and was thus chosen as an extreme wavelength range^22^. After irradiation, the films were investigated by FTIR measurements. The overlaid FTIR absorption spectra shown in Fig. 2 are recorded after 24, 48 and 672 h of irradiation for pp–GT thin films fabricated at 50 W RF power. To test the effect of available oxygen, selected experiments were repeated using a quartz cover to limit the amount of oxygen reaching the sample (denoted as ‘oxygen-poor’ conditions).

A broad absorbance band emerged at 3416 cm^−1^ in the non-irradiated pp–GT spectrum that is considered to be the O–H stretching vibration of residual water^23^. This is further supported by the absence of a C–O vibration. Absorptions corresponding to C–H bending vibrations (between 1300 and 1466 cm^−1^) decreased in magnitude and broadened with higher input RF power. Likewise, skeletal C–C vibrations between 947 and 1161 cm^−1^ vanished completely. A peak emerged at 1707 cm^−1^, indicating the presence of carbonyl groups (C=O stretch), which were presumably formed from the reactions of radical intermediates with residual molecular oxygen and subsequent decompositions^23^. In general, polymer materials containing C=O (and –OH) groups are characterized by a high-dielectric constant, because of their high polarizability and lower hydrophobicity. The CA investigations in this study revealed that pp–GT films deposited at lower RF power were indeed less stable when in contact with common processing solvents, such as acetone or alcohol. The absence of the C=O moiety may render pp–GT more stable when in contact with polar solvents.

The nature of irradiation and oxygen content had significant effects on the photostability of the pp–GT thin films. The UV-spectra of the freshly fabricated films reached ~400 nm, thus light absorption was achieved under all irradiation conditions. Upon irradiation with UV–A light in an oxygen-rich environment, strong and broad bands rapidly emerged at approx. 3400 cm^−1^ and 1700 cm^−1^, respectively. The changes in intensities decreased during the course of the irradiation, suggesting ‘inner-filter’ effects or saturation of the surface by the photodegradants. The newly formed peaks were assigned to oxygen-containing functional groups, in particular O–H (hydroxyl and carboxyl groups) and C=O stretching vibrations (carbonyl and carboxyl groups). Peak broadening furthermore occurred in the region below 1500 cm^−1^, possibly due to the emergence of C–O stretching vibrations. The observed changes thus suggest photooxidation of the pp–GT thin films upon light exposure. Similar observations have been reported for ethylene-propylene^24^ and styrenic polymers^25^. The intensity and shape of the C–H stretching vibration around 2900 cm^−1^ remained largely unchanged, suggesting that the carbon-hydrogen backbone of the polymer stayed largely intact. When exposed to UV–B light, the same changes were in principle observed in the IR-spectra, although more rapidly. In contrast to UV–A exposure, the intensity of the C–H stretching vibrations around 2900 cm^−1^ dropped significantly. This behavior can be explained by the stronger absorption of the polymeric film in the UV–B range and hence increased photodegradation. In addition, the initial photooxidants formed, i.e. carbonyl-containing compounds, absorb in the same region, thus enabling further oxidation to carboxylic acid or other photodegradants^26^. Rapid degradation was again observed upon exposure to UV–C light. The generation of oxygen-containing functional groups was somewhat retarded, whereas a sharp drop and broadening of the C–H stretching vibration at approx. 2900 cm^−1^ was found. These observations suggest, next to photooxidations, a range of alternative degradation processes such as rearrangements, chain scissions and crosslinking reactions^27^,^28^.

Selected pp–GT thin films have also been irradiated under oxygen-poor conditions by covering the films with a suitable quartz plate (transmission approx. >200 nm). UV–A and UV–C light were chosen to represent the two extreme light conditions. No changes were observed in the FTIR spectra of UV–A irradiated pp–GT thin films compared to the non-irradiated films (not shown). The photostability observed suggests that the power of the UV–A light was insufficient to form significant amounts of reactive species that can react with residual molecular oxygen within the film. In contrast, when the pp–GT thin films were photolyzed with highly energetic UV–C light, degradation was observed, although the process was significantly slower than in an oxygen-rich environment. The FTIR spectrum taken after 672 h of exposure suggested significant photooxidation even under ‘oxygen-poor’ conditions. Thus, UV–C light is able to generate and maintain reactive intermediates that rapidly react with residual oxygen present in the polymeric film. The sharp drop in the C–H vibration region suggests that non-oxidative photolyses pathways dominate the overall degradation process.

### Surface morphology of UV irradiated pp–GT films

**Figure 3:**
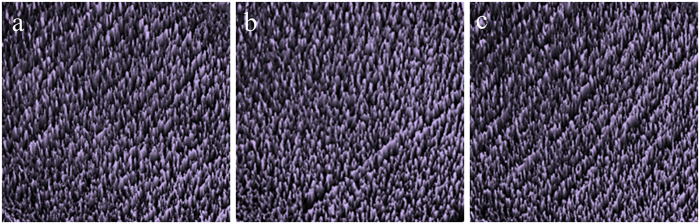
Representative AFM images of pp–GT thin films (50 W) after (**a**) UV–A, (**b**) UV–B and (**c**) UV–C irradiation for 24 h.

Figure 3 shows representative AFM images collected on the pp–GT samples fabricated at 50 W RF power that have been irradiated with UV–A, UV–B and UV–C light for 24 h under oxygen–rich conditions. The pp–GT thin films (as deposited) are generally smooth, uniform and defect-free, exhibiting consistent morphology across the film samples^10^. Average roughness values for non-irradiated samples are approximately 0.3 nm^10^. However, the UV-illuminated samples showed an increase in roughness with prolonged exposure time. An increase of the intensity of UV irradiation also increased the roughness (Table 1). This observation confirms that chemical changes due to photochemical transformations occurred at the polymer surface but required initiation by high-energy light.

The mechanical properties of pp–GT thin films were also investigated using a load–partial unload technique. A series of indentations were made and the hardness of pp–GT thin film samples was found to decrease with increasing both the UV–illumination time and the energy of UV irradiation. Table 1 shows hardness data collected for polymer thin films deposited at 50 W RF power. Hardness values for films fabricated under other deposition powers displayed similar behavior, with films fabricated at lower RF power being more susceptible to UV-induced degradation.

### Wetting behavior of UV irradiated pp–GT

A contact angle hysteresis (CAH) approach^23^ was used to investigate the wetting behavior of pp–GT films fabricated at 50 W and irradiated with UV–A, UV–B and UV–C under oxygen-rich conditions. The results (advancing CA *θ*_*A*_, receding CA *θ*_*R*_, and work of spreading *W*_*S*_) are listed in Table 2. In line with FTIR and AFM data, an increase in irradiation time and energy of the UV light resulted in lower values of advancing and receding CAs. The relatively low CAH for 24 h UV–irradiated films suggests higher chemical homogeneity and a lower degree of photodecomposition in these films. CAH subsequently increased as the irradiation time increased (Table 2), which is attributed to the higher degree of chemical changes in combination with a reduction of cross-linking in the pp–GT films^29^ and the presence of polar groups, that subsequently led to a more chemically-heterogeneous surface. An increase in surface roughness, as indicated by the AFM study, may also contributed to the observed changes in surface wetting.

The wettability of each solution on the pp–GT surface can also be quantified by computing the work of spreading *W*_*S*_^30^. It shows the extent to which the liquid will adhere to the surface relative to itself and, hence, indicates the ability of a liquid drop to stick to the solid surface^31^. The pp–GT thin films were characterized by increased wettability with increased irradiation time and intensity of UV light (Table 2). This confirms the continuous formation of polar functional groups through photooxidation during the course of the irradiation^28^,^32^.

## Discussion

Photostability of polymer thin films is a crucial parameter, especially for exterior surfaces or applications as OPV devices that are exposed to solar radiation. Typically these films are exposed to UV light via sunlight. Solar radiation reaching the surface of the earth is characterized by wavelengths from approximately 295 up to 2500 nm. The solar radiation classified as UV–B (280–315 nm) has an energy of 426–380 kJ mol^−1^ and may thus induce bond cleavages^28^. However, the higher energetic part of UV–B (280–295 nm) is filtered by the stratosphere and does not reach the earth’s surface. UV–A light (315–400 nm) has energy between 389 and 300 kJ mol^−1^ and is thus less harmful for organic materials than UV–B^28^. Likewise, visible (400–760 nm) and infrared (760–2500 nm) radiation do not possess sufficient energy amounts to cause significant chemical changes. UV radiation below 295 nm (UV–C) causes significant degradation but does not occur under natural conditions^33^,^34^. However, a high dose of UV radiation (UV–A or UV–B) can have detrimental effects on the properties of these polymers. This degradation generally occurs due to C–C bond scission^35^ resulting not only in chain scission but also chemical changes of side groups. In the presence of oxygen, photooxidation processes with triplet oxygen (^3^O_2_) can furthermore occur, resulting in oxygenation of the polymer and generation of oxygen-containing functional groups^27^,^30^. Likewise, photosensitized oxygenations involving singlet oxygen (^1^O_2_) may operate^36^,^37^, especially in the presence of suitable sensitizing agents. While undesired for polymers, these reactions are useful in organic synthesis with sunlight^38^,^39^. Photostable polymers are also often used as solid supports for photooxygenation reactions^40^.

Using absorption spectroscopy, it has been previously demonstrated that pp–GT thin films possessed similar absorption spectra when deposited and after prolonged storage under ambient conditions^10^, a behavior that was similar to that for other plasma polymers fabricated from terpenes^41^. For instance, for plasma polymers from terpinen-4-ol, prolonged exposure to air under room temperature (~20 °C) led to an insignificant change in optical properties and surface chemistry, with the bulk of the changes occurring within the first few days after deposition, followed by stabilization of these properties for the duration of the study^41^. It is therefore likely that the significant changes in optical and chemical properties in pp–GT after UV exposure are a result of photodegradation^42^,^43^. It is also important to note that changes in the rate of decomposition may be related to light-absorption and thus filtering by certain photodegradants.

In general, polymers can degrade by a variety of different mechanisms^44^. Of these, oxidation, i.e. the reaction with molecular oxygen, in the absence or presence of light, occurs naturally and can significantly reduce the lifespan of polymer films^28^,^45^,^46^. Commonly, materials subjected to oxygen are degraded much faster in the presence of radiation than in its absence and vice versa. For instance, organic polymer-based encapsulating strategies, such as those based on ethylene vinyl acetate (EVA) are promising in that they offer long-term weather resistance^47^, however under UV irradiation and oxygen-rich conditions EVA films have been shown to undergo rapid photooxidation and incorporation of ketone structures, which can be further degraded. This may not only affect the properties of the polymer, but also the adhesion strength, in particular under damp heat conditions and prolonged exposure time.

**Figure 4:**
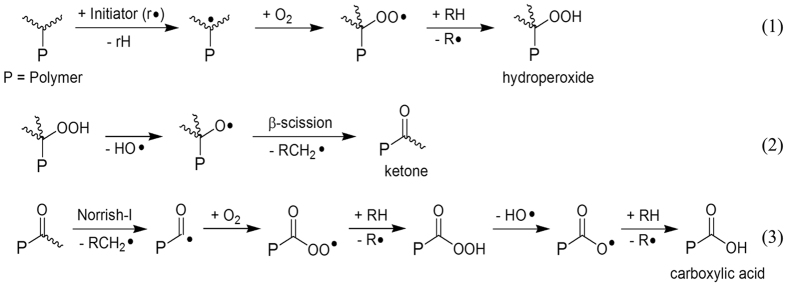
General mechanism of photooxidation of pp–GT films (wavy lines represent possible bonds within the polymer).

When pp–GT films were subjected to UV irradiation under oxygen–rich conditions, rapid photooxidation was observed. In contrast to other polymers, e.g. polystryrene^28^, no visible changes such as yellowing or gradual embrittlement could be observed. The presence of oxygen-containing functional groups and thus of chemical changes was unambiguously demonstrated by FTIR analysis (Fig. 2)^26^,^48^. In contrast to thermal oxidations or autooxidation^46^, photooxidation is not limited to activated positions, e.g. reactive allylic positions within physically-incorporated monomer units. Instead, the degradation pathways for pp–GT films depended on the energy of the light^49^,^50^. Consequently, in addition to autooxidation via reactions involving allylic hydroperoxides^46^ or alkoxy-radicals^26^,^28^, additional degradation pathways are possible. As an example, the excess excitation energy from UV-irradiation may have initiated the cleavage of a C–H bond in tertiary alkyl-sidechains of the polymer [equation 1 (Fig. 4)]. Instant oxygen trapping, followed by β-scission furnished the corresponding ketone-containing degradants [equation 2 (Fig. 4)]^28^,^50^. Subsequent Norrish-I cleavage followed by reaction of the acyl radical with molecular oxygen and successive follow-up reactions produced the carboxylic acid functions detected by FTIR [equation 3 (Fig. 4)].

UV irradiation has a significant impact on the photodegradation of pp–GT films. Upon irradiation with UV–A light, the initially formed hydroperoxides [equation (1)] readily decomposed to produce carbonyl groups^28^,^51^. Photolysis with even more energy-rich UV–B light showed faster oxidation and a significant drop in the intensity of the C–H FTIR-vibration peak (Fig. 2). UV–B light is able to activate the intermediary formed carbonyl compounds that are the main products of photodegradation^50^, thus opening Norrish-type cleavage pathways (equation 3). Rapid photooxidation was also observed upon irradiation using UV–C light. This light is capable of cleaving a variety of chemical bonds including residual C=C bonds. The intermediates formed were subsequently trapped by oxygen to yield oxygen-containing functional groups. In oxygen-poor conditions, the pp–GT films remained stable when exposed to UV–A light. However, photooxidative and in particular other decompositions were still observed with UV–C light, thus highlighting the importance of homogeneous bond cleavages for this energy-rich light.

The photooxidation stability of future polymer films can be enhanced by changing their chemical composition, e.g. reducing the content of residual C=C- moieties, and reducing the portion of unreacted monomer that may be incorporated into the matrix of the film during plasma polymerization. Higher cross-linking typically observed in plasma polymers deposited at higher input power may also positively contribute to photochemical stability of the film. Incorporation of nanostructures or UV absorber substances into the matrix of the film may also afford some protection against the harmful effects of UV radiation^52^.

## Conclusions

This study demonstrated that pp–GT thin film encapsulation deposited directly onto PCPDTBT:PC_70_BM solar cells without any additional UV-curable epoxy resin significantly slowed down degradation of the device performance over 80 days of observation. These results, coupled with previously reported ambient and thermal stability of up to 200 °C and excellent adhesion of thus-fabricated polymers, provide further evidence for the use of pp–GT thin films as potential barrier coatings for encapsulation of OPV devices. This work also demonstrated that, like most other organic polymers, pp–GT is subject to degradation via photooxidation, with the extent of degradation dependent on both the intensity of the light and the abundance of oxygen. When irradiated with lower-energy UV–A light, the presence of oxygen from the ambient atmosphere played a significant role, where the use of a quartz mask limiting the supply of oxygen notably slowed down the ageing of the polymer. On the other hand, even with the use of the mask, photochemical degradation as a result of exposure to high-energy UV–C light proceeded relatively quickly, supporting additional non-oxygen-demanding degradation pathways. It should be noted that the irradiation conditions used in this study were greatly exaggerated in comparison to the ambient operating conditions these films would encounter in real outdoor applications. Furthermore, being a function of fabrication conditions, the stability may be further improved through optimization of the fabrication process, e.g. by depositing the films at higher RF power or co-polymerising with other monomers^28^,^53^.

## Methods

### Preparation of thin films

Polymer thin films were deposited using a custom designed PECVD reactor on high quality glass slides. The cylindrical RF plasma chamber was 0.75 m in length, with an inner diameter of 0.055 m (approximate volume of 0.018 cm^3^). Two removable caps at the ends of the chamber contained inlets for processing gas (typically Ar) and vacuum gauge, and an outlet to a rotary vacuum pump. Optimization of the deposition conditions was performed by altering the distance between the monomer outlet and the electrodes, the distance between the electrodes, deposition pressure, and RF power to obtain transparent, smooth and uniform thin films across the substrate. For all depositions performed in this study, which took place without the use of processing gas, a working pressure of 100–150 mT was used. Prior to each deposition, the chamber was flushed with argon. An RF generator operating at 13.56 MHz was used to deliver the desired RF energy into the chamber using capacitive coupling by means of external copper electrodes placed 0.13 m from the monomer inlet and 0.1 m apart. The RF input power was varied between 10 W and 75 W. Above 75 W, the strength of the field began to interfere with sensitive electronics in the proximity of the system (such as the vacuum gauge) and overheat the matching network. To ensure accurate pressure readings and correct operation of the matching network, 75 W was the highest power used in this study.

Glass substrates (25 mm × 75 mm) were thoroughly cleaned using extran, an ultrasonic bath of distilled water, and rinsed with isopropanol prior to deposition. Films were deposited at room temperature, with 10 mL of the monomer used for every deposition. Since this monomer is volatile under ambient temperature (boiling point of 181–183 °C at 760 mm Hg; vapor pressure of 1.075 mm/Hg at 25 °C; vapor density of 4.7), monomers were gradually delivered into the chamber without the use of external heating. The rate of the monomer flow was controlled to be ~1.5–1.6 cm^3^ min^−1^.

### OPV device characterization

**Figure 5:**
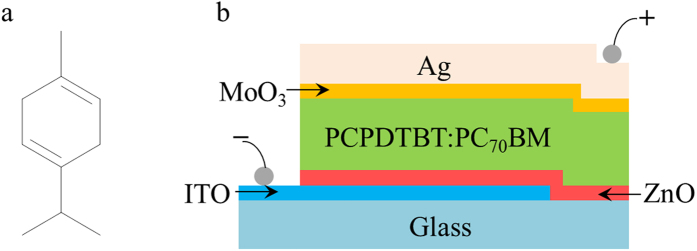
(**a**) Chemical structure of 1-isopropyl-4-methyl-1,4-cyclohexadiene (C10H16). (**b**) Schematic diagram of OPV device used in this study.

The effect of the pp–GT encapsulation on solution-processed OPV devices based on a blend of electron donor material poly[2,1,3-benzothiadiazole-4,7-diyl[4,4-bis(2-ethyl- hexyl)-4H-cyclopenta[2,1-b:3,4-b′]dithiophene-2,6-diyl]] (PCPDTBT) and electron acceptor material [6,6]-phenyl C_70_ butyric acid methyl ester (PC_70_BM) was studied. PCPDTBT and PC_71_BM were purchased from Chemscitech Inc., and used without further modification. Pre-patterned indium tin oxide (ITO) substrates (12 mm × 12 mm) were purchased from Lumtec. The inverted device structure ITO/ZnO/PCPDTBT:PC_71_BM/MoO_3_/Ag (Fig. 5b) was prepared following the procedure outlined in ref. 21, resulting in the device area of 0.13 cm^2^. To produce encapsulated samples, devices were coated with 200 nm pp–GT fabricated at 25 W.

QEX10 spectral response system (PV measurements, Inc.) was used to measure external quantum efficiency (EQE). Measurements of current density–voltage (*J*–*V)* characteristics were performed using an IV5 solar cell *I*–*V* testing system (PV measurement, Inc.) and a Keithley 2400 source meter under illumination power of 100 mW cm^−2^ generated by an AM 1.5 G solar simulator (Oriel model 94023 A). OPV device efficiency and *J–V* properties of as-fabricated and encapsulated devices were measured for a period of 1920 h (80 days).

### Photostability study

A top irradiation chamber photoreactor (Luzchem LZC-1) equipped with fluorescent tubes (8 × 8 W) of different wavelengths (λ = 254, 300 ± 25 and 350 ± 25 nm) was used in this study. The internal irradiation chamber had internal dimensions of 30.48 cm (w) × 30.48 cm (d) × 21.59 cm (h). A build-in fan and exhaust stabilized the temperature to about 3–4 °C above room temperature. For UV–A (350 ± 25 nm) and UV–B (300 ± 25 nm) irradiations, 8 W fluorescent tubes from Southern New England Ultraviolet Company (model RPR-3000 Å and RPR-3500 Å, respectively) were used. Philips UVC (germicidal) lamps (model TUV 8 W FAM) were used for UV–C (254 nm) irradiations. After the irradiation, pp–GT samples were examined by variable angle spectroscopic ellipsometer (model M-2000, J. A. Woollam Co. Inc.; within 200–1000 nm spectral range and at angles of incidence φ = 55°, 60°, and 65°), ATR-FTIR (Perkin Elmer Spectrum 100 FTIR spectrometer, within 4000–500 cm^−1^ region at a resolution of 1 cm^−1^), AFM (NT-MDT NTEGRA Prima, in tapping mode, using NSC05 cantilevers), and CA measuring system (KSV 101).

## Additional Information

**How to cite this article:** Bazaka, K. *et al*. Photostability of plasma polymerized γ-terpinene thin films for encapsulation of OPV. *Sci. Rep.* **7**, 45599; doi: 10.1038/srep45599 (2017).

**Publisher's note:** Springer Nature remains neutral with regard to jurisdictional claims in published maps and institutional affiliations.

## Figures and Tables

**Table 1:** Average roughness and hardness of non-irradiated and UV–A, UV–B and UV–C irradiated pp–GT thin films (films fabricated at 50 W).

	Irradiation time (h)		
	24	48	672
*Average roughness, R*_*a*_ (*nm*)			
Pre-irradiation		0.25 ± 0.08	
UV–A	2.24 ± 0.09	2.84 ± 0.13	3.85 ± 0.25
UV–B	2.57 ± 0.08	2.98 ± 0.15	3.91 ± 0.18
UV–C	2.81 ± 0.11	3.18 ± 0.19	4.41 ± 0.27
*Hardness (GPa*)			
Pre-irradiation		0.51 ± 0.01	
UV–A	0.51 ± 0.03	0.47 ± 0.02	0.48 ± 0.02
UV–B	0.47 ± 0.02	0.44 ± 0.01	0.41 ± 0.03
UV–C	0.42 ± 0.03	0.36 ± 0.03	0.31 ± 0.04

**Table 2:** Wettability parameters of pp–GT thin films fabricated at 50 W and irradiated with UV–A, UV–B and UV–C.

UV irradiation	θ_A_, °	θ_R_, °	CAH, °	W_S_ (mJ·m^−2^)
UV–A (24 h)	72.8	28.2	44.7	−50.7
UV–B (24 h)	63.7	26.0	37.7	−40.1
UV–C (24 h)	49.8	19.5	30.3	−25.5
UV–A (672 h)	69.0	24.0	45.1	−46.2
UV–B (672 h)	58.8	19.1	39.7	−34.6
UV–C (672 h)	42.3	10.8	31.5	−18.8
